# Serum concentrations of symmetric dimethylarginine and creatinine in cats with kidney stones

**DOI:** 10.1371/journal.pone.0174854

**Published:** 2017-04-06

**Authors:** Jean A. Hall, Maha Yerramilli, Edward Obare, Jun Li, Murthy Yerramilli, Dennis E. Jewell

**Affiliations:** 1 Department of Biomedical Sciences, College of Veterinary Medicine, Oregon State University, Corvallis, Oregon, United States of America; 2 IDEXX Laboratories, Inc., One IDEXX Drive, Westbrook, Maine, United States of America; 3 Pet Nutrition Center, Hill's Pet Nutrition, Inc., Topeka, Kansas, United States of America; Hospital Universitario de la Princesa, SPAIN

## Abstract

Serum concentrations of symmetric dimethylarginine (SDMA) correlate with renal function in cats and SDMA has been shown to be a more reliable and earlier marker for chronic kidney disease (CKD) compared with serum creatinine (Cr). Calcium oxalate uroliths tend to develop in mid-to-older aged cats and kidney stones may cause a reduction in renal function with increased SDMA, but normal serum Cr. The purpose of this retrospective study was to determine if cats with kidney stones had increased serum SDMA concentrations, and whether SDMA increased earlier than serum creatinine concentrations. Cats in the colony with kidney stones diagnosed between August 2010 and December 2015 (n = 43) were compared with healthy geriatric cats (n = 21) without kidney stones. Serum SDMA concentrations were determined by liquid chromatography-mass spectrometry and serum Cr concentrations were determined by enzymatic colorimetry. Cats with kidney stones were diagnosed antemortem by radiographic imaging (n = 12) or by postmortem necropsy (n = 31). Retrospectively, serum SDMA was found to be increased above the upper reference limit in 39 of 43 cats with kidney stones. Serum Cr was increased above the upper reference limit in 18 of 43 cats; 6 of these 18 cats had terminal azotemia only. The mean time that serum SDMA was increased before serum Cr was increased was 26.9 months (range 0 to 60 months). Kidney stones were composed of calcium oxalate in 30 of 34 cats. The lifespan for cats with kidney stones (mean, 12.5 years; range, 6.1 to 18.1 years) was shorter (*P* < 0.001) than for control cats (mean, 15.2 years; range, 13.0 to 17.2 years), suggesting that non-obstructive kidney stones have an effect on mortality rate or rate of CKD progression. In conclusion, if SDMA concentrations are elevated in mid-to-older aged cats, further imaging studies are warranted to check for the presence of kidney stones.

## Introduction

Beginning in the mid-1980s, a noticeable increase in the frequency of calcium oxalate uroliths occurred in cats concomitant with a decrease in the frequency of struvite uroliths [1]. In 2002, approximately 55% of the 95,000 feline uroliths submitted to the Minnesota Urolith Center were composed of calcium oxalate [1]. More recently (2007), the frequency of feline calcium oxalate uroliths declined to 41%, perhaps associated with reformulation of adult maintenance diets to minimize risk factors for calcium oxalate crystalluria [1]. These finding are similar to the percentage of feline calcium oxalate uroliths (40%) reported from submissions to the Gerald V. Ling Urinary Stone Analysis Laboratory [2]. The increase in calcium oxalate urolithiasis in cats has been associated with a parallel increase in the occurrence of these uroliths in the upper urinary tract (kidneys and ureters) [1].

Calcium oxalate uroliths tend to develop in mid-to-older aged cats [3]. Factors associated with increased risk of calcium oxalate urolithiasis are feeding urine-acidifying diets, feeding a single brand of cat food, being kept indoors only, and being of the Persian breed [3]. It has been reported that calcium oxalate urolith formation occurs when urine is supersaturated with calcium and oxalate [4], although all healthy cats have urine that is supersaturated with calcium and oxalate. In one study, urine relative supersaturation with calcium oxalate was higher in healthy cats fed an acidifying diet, supporting a pH effect on urinary saturation with calcium oxalate, however, there were no significant differences in urinary excretion of calcium, oxalate, or citrate [5]. Thus, it is likely that other risk factors, perhaps dietary, genetic, or idiosyncratic, are operative.

We have previously reported that serum concentrations of symmetric dimethylarginine (SDMA) were increased in cats with chronic kidney disease (CKD), and that SDMA concentrations increased earlier than serum creatinine concentrations in cats with CKD [6]. In that study, SDMA concentrations were increased in three azotemic and two nonazotemic cats with calcium oxalate nephrolithiasis out of 21 cats with CKD [6]. We have also shown that serum SDMA correlates (*r* = –0.79) with glomerular filtration rate (GFR) in cats [6, 7]. Furthermore, serum SDMA concentrations are not affected by lean body mass in cats [7]. Thus, cats with calcium oxalate nephrolithiasis could have IRIS stage 1 CKD with increased SDMA and normal serum Cr.

The purpose of this study was to retrospectively measure serum SDMA concentrations in cats with previously diagnosed kidney stones, provided banked serum samples were available for measurement, to determine if cats with kidney stones have increased serum SDMA concentrations. If true, increased serum SDMA concentration would be an indication to perform radiographic imaging to check for the presence of kidney stones in cats, before kidney function declines further and serum Cr concentrations increase.

## Methods

### Animals and study design

All study protocols and this study were reviewed and approved by the Institutional Animal Care and Use Committee, Hill’s Pet Nutrition, Inc., Topeka, KS (Permit Number: 678). Each cat had an annual physical examination, CBC, serum biochemical analyses, urinalysis, and urine culture if indicated by the urinalysis results. In addition, after 2010, serum was frozen at -70°C and banked for retrospective analyses. Cats were housed individually or in groups and allowed exercise in indoor runs. Cats had access to natural light that varied with seasonal changes. All cats were provided with regular opportunities to exercise, with access to toys. All cats were owned by the commercial funders of this research or their affiliates, who gave permission for them to be included in this study.

All cats had been fed many types of commercial and non-commercial foods of varying nutrient compositions, including dry and canned cat foods, in palatability studies. All foods met the requirements established by the Association of American Feed Control Officials for complete and balanced pet foods for adult cats.

Cats with kidney stones came from the colony of over 400 domestic short hair cats. Cats in the colony ranged in age from 1 to 19 years with approximately 25% of cats > 10 years. All cats with kidney stones diagnosed from August 2010 to December 2015 were included (n = 43). Mean age of cats was 11.7 years (range, 4.7 to 18.1 years) at the time of diagnosis. There were 22 ovariohysterectomized females and 21 neutered males.

A group of healthy geriatric cats (n = 21) was selected from the same colony. Criteria for inclusion were age > 10 years, requirement of 3 normal GFR tests, 3 normal sCr concentrations, and 3 urine specific gravity (USG) measurements > 1.035 over a 6-month period. In addition, these cats lacked historical or physical evidence of confounding disease at the time of inclusion, and had banked serum samples available for determination of SDMA concentrations. Mean age of healthy geriatric cats was 11.7 years (range, 10.2 to 13.1 years). There were 12 ovariohysterectomized females and 9 neutered males. Kidney stones were not detected by subsequent radiographic imaging or postmortem necropsy.

### Serum biomarkers

Retrospective data was used to document SDMA concentrations in cats with kidney stones from serum stored in serum banks. Serum creatinine (Cr) and urea nitrogen concentration were determined by enzymatic colorimetric methods (Roche Diagnostics, Cobas 6000 series, c501 module, Indianapolis, IN). Reference ranges for serum Cr (0.7–2.1 mg/dL) and urea nitrogen (15.0–31.0 mg/dL) in other adult cats were previously established for this in-house laboratory. SDMA concentrations were determined using liquid chromatography-mass spectroscopy (LC-MS) as previously described [6]. The upper limit of the reference interval for SDMA was determined by a commercial laboratory (IDEXX Laboratories, Inc., Westbrook, ME) in healthy cats to be < 14 μg/dL.

### Urinalysis

Urine specific gravity was determined using a refractometer. Urine protein to creatinine ratio calculations were determined as previously reported [6] and expressed as mg/dL protein:mg/dL creatinine.

### Statistical analysis

Demographic data for cats with kidney stones and healthy cats without kidney stones are reported. Parameters assessed included method of diagnosis of kidney stones (radiographic imaging or postmortem necropsy), age at diagnosis of kidney stones, gender, and stone analysis. In those cats with kidney stones diagnosed antemortem using radiographic imaging, serum concentrations of SDMA, Cr, and urea nitrogen, and USG and UPC ratio at the time of diagnosis are reported, with follow up at death or last assessment if still alive. In those cats with kidney stones diagnosed at postmortem necropsy, serum concentrations of SDMA, Cr, and urea nitrogen, and USG and UPC ratios at death are reported. Percentages of cats with normal or increased serum concentrations of SDMA, Cr, and urea nitrogen, and normal or increased USG and UPC ratio were determined. In addition, the approximate time (months) that serum SDMA was increased before Cr increased or the cat died was calculated. Data are reported as mean (median; range) in Table 1.

**Table 1:** Demographic data, mean (median; range), are summarized for healthy cats, cats with kidney stones diagnosed antemortem by radiographic imaging, and cats with kidney stones diagnosed postmortem at necropsy.

	Healthy Cats (N = 21)	Cats with Kidney Stones Diagnosed Antemortem by Radiographic Imaging (N = 12)					Cats with Kidney Stones Diagnosed Postmortem at Necropsy (N = 31)	
Variable		At the time of diagnosis	*P* Value^a^	% Above or below normal^b^	At the time of death (N = 6) or last assessment if alive (N = 6)	% Above or below normal^b^	At the time of death	% Above or below normal^b^
Age (years)	11.7 (11.9; 10.2–13.1)	8.8 (7.3; 4.7–15.1)			10.6 (8.8; 6.9–16.7)		12.9 (12.7; 6.1–18.1)	
SDMA (μg/dL) Normal < 14 μg/dL	9.9 (9.8; 7.3–12.4)	16.7 (15.7; 13.1–25.1)	<0.01	11/12 (92%)	31.5 (18.5; 12.5–162.2)	11/12 (92%)	23.1 (16.3; 8.5–100.0)	23/31 (74%)
Cr (mg/dL) Normal < 2.1 mg/dL	1.2 (1.2; 0.7–1.6)	1.7 (1.6; 1.4–2.9)	<0.01	2/12 (17%)	3.3 (1.7; 1.0–17.9)	4/12 (33%)	2.7 (1.6; 0.4–12.2)	11/31 (35%)
Urea nitrogen (mg/dL) Normal < 31.0 mg/dL	19.7 (19.4; 16.7–26.0)	29.7 (28.7; 23.2–40.7)	<0.01	3/12 (25%)	51.7 (35.8; 18.2–229.5)	8/12 (67%)	49.8 (31.0; 22.0–175.3)	15/29 (52%)
USG Normal > 1.035	1.059 (1.062; 1.039–1.078)	1.040 (1.042; 1.019–1.055)	<0.01	10/12 (83%)	1.033 (1.035; 1.007–1.056)	7/12 (58%)	1.027 (1.030; 1.008–1.051)	8/27 (30%)
UPC ratio Normal < 0.4	0.2 (0.2; 0.1–0.3)	0.19 (0.17; 0.10–0.42)	NS	1/11 (9%)	0.22 (0.20; 0.08–0.40)	0/8 (0%)	0.31 (0.29; 0.05–0.71)	6/19 (32%)
SDMA increased before Cr (months)	0.0 (0.0; 0.0–0.0)				33.7 (34.0; 0.0–60.0)		24.0 (18.5; 0.0–58.0)	

^a^ Compared with healthy cats.

^b^Percentage of cats assessed that had values above the upper or below the lower limit of the reference interval (shown in variable column).

Statistical analyses were performed using SAS version 9.4 (SAS Institute, Cary, NC). To test the hypothesis that serum concentrations of SDMA, Cr, and urea nitrogen, and USG and UPC ratios in healthy geriatric cats were different from cats with kidney stones diagnosed antemortem using radiographic imaging, the SAS procedure PROC NPAR1WAY was used to compare these two groups by the Mann-Whitney test. This test has the advantage over parametric tests in that it does not require an assumption of normality [8]. A *P*<0.05 was considered statistically significant.

## Results

### All cats with kidney stones

Cats with kidney stones (n = 43) were diagnosed antemortem by radiographic imaging (n = 12) or by postmortem necropsy (n = 31). Six cats diagnosed antemortem by radiographic imaging are still alive; six are deceased. Kidney stones noted antemortem by radiographic imaging were not found at necropsy in three cats.

Stone analysis was performed at the Minnesota Urolith Center (University of Minnesota, St. Paul, MN) in cats with stones found at necropsy (n = 34). Kidney stones were composed of calcium oxalate (n = 30; 5 had miscellaneous material reported also), 100% calcium phosphate apatite form (n = 2), or miscellaneous material only (n = 2).

Serum SDMA was increased (≥ 14 μg/dL) at some point prior to death in 39 of 43 cats with kidney stones. In 4 cats, all deceased, serum SDMA was always normal (< 14 μg/dL). Using the diagnosis of kidney stones antemortem by radiographic imaging or the finding of kidney stones postmortem at necropsy as the gold standard, sensitivity (91%), specificity (100%), PPV (100%), and NPV (84%) were calculated for serum SDMA concentration. Serum Cr was increased at some point prior to death in 18 of 43 cats; 6 of the 18 cats had terminal azotemia only. The mean time that serum SDMA was increased before serum Cr was increased in these 39 cats was 26.9 months (range, 0 to 60.0 months). Serum urea nitrogen was increased at some point prior to death in 25 of 43 cats; 10 of the 25 cats had terminal elevation only.

Urine specific gravity was > 1.035 in 15 of 39 cats and UPC ratio was > 0.4 in 6 of 27 cats with kidney stones at the time of death or last assessment if alive.

Cats that died with kidney stones (n = 37) lived for mean, 12.5 years (range, 6.1 to 18.1 years; SEM 0.44 years). The six cats with kidney stones that are still alive were not included in this age analysis. Healthy control cats (n = 21) had longer lifespans (*P* < 0.001) living for mean, 15.2 years (range, 13.0 to 17.2 years; SEM 0.58 years). There are 5 healthy control cats still alive; these cats were included using their current age as age of death.

### Cats with kidney stones diagnosed antemortem

**Fig 1:**
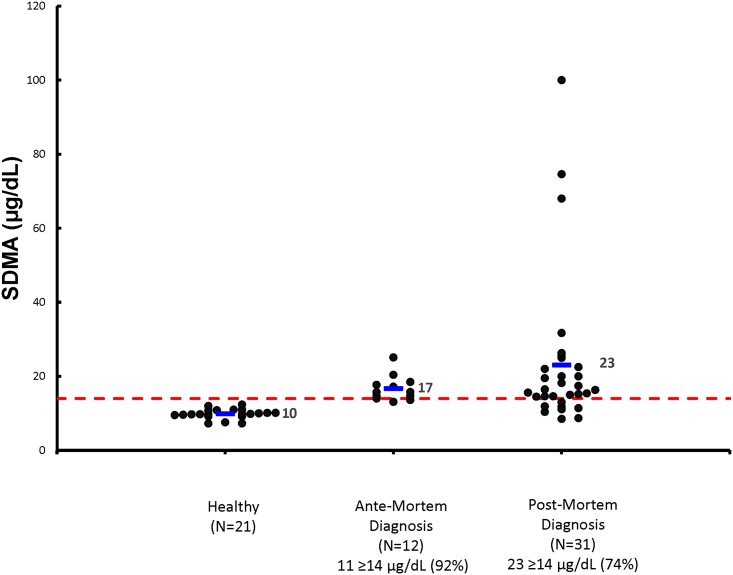
Serum SDMA concentrations are illustrated for healthy cats, cats with kidney stones diagnosed antemortem at the time of diagnosis, and cats with kidney stones diagnosed postmortem at the time of death. The dashed horizontal red line represents the upper limit of the reference interval for serum SDMA concentration (< 14 μg/dL). Individual cats in each group are represented by a black dot. The horizontal blue line represents the mean serum SDMA concentration for the group. The number and percent of cats ≥14 μg/dL in each group are also indicated.

**Fig 2:**
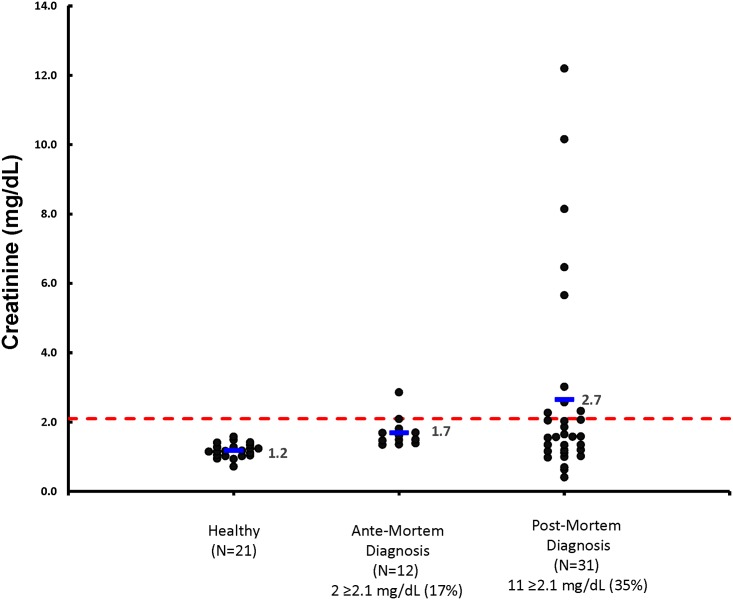
Serum Cr concentrations are illustrated for healthy cats, cats with kidney stones diagnosed antemortem at the time of diagnosis, and cats with kidney stones diagnosed postmortem at the time of death. The dashed horizontal red line represents the upper limit of the reference interval for serum Cr concentration (2.1 mg/dL). Individual cats in each group are represented by a black dot. The horizontal blue line represents the mean serum Cr concentration for the group. The number and percent of cats ≥ 2.1 mg/dL in each group are also indicated.

The 12 cats diagnosed with kidney stones antemortem by radiographic imaging lived for several months after diagnosis (mean, 21.5 months; range 5.0–47.0 months). Demographic data, mean (median; range) are summarized in Table 1 for serum SDMA, Cr, and urea nitrogen concentrations, and for USC and UPC ratios, both at the time of diagnosis and at the time of death or last assessment if still alive. At the time of diagnosis, serum SDMA concentrations were increased in 92% of these cats (Fig 1), serum urea nitrogen in 25% of cats, and serum Cr in 17% of cats (Fig 2). The USG was > 1.035 in 10 out of 12 cats assessed, and the UPC ratio > 0.4 in 1 out of 11 cats assessed.

None of the healthy control cats (n = 21) had kidney stones diagnosed antemortem by subsequent radiographic imaging (n = 5) or postmortem necropsy (n = 16). Serum SDMA, Cr, and urea nitrogen concentrations were all within the normal reference interval. The USG was > 1.035 and UPC ratio ≤ 0.3 in all control cats.

Compared with healthy control cats, cats with kidney stones at the time of diagnosis had increased serum SDMA (*P* < 0.001), Cr (*P* < 0.01), and urea nitrogen (*P* < 0.001) concentrations. The USG was also lower (*P* < 0.001) in cats with kidney stones at the time of diagnosis compared with healthy control cats. The mean time that serum SDMA was increased before serum Cr was increased in these 12 cats was 33.7 months (range, 0 to 60.0 months).

### Cats with kidney stones diagnosed postmortem

Demographic data, mean (median; range) are summarized in Table 1 for the 31 cats with kidney stones diagnosed postmortem. At the time of death, serum SDMA concentrations were increased in 74% of these cats (Fig 1), serum urea nitrogen in 52% of cats, and serum Cr in 35% of cats (Fig 2). The USG was > 1.035 in 8 out of 27 cats, and the UPC ratio > 0.4 in 6 out of 19 cats assessed. Serum SDMA concentration had been increased on sampling dates prior to death in 4 of 8 cats with normal SDMA concentrations at death. The mean time that serum SDMA was increased before serum Cr was increased in these 31 cats was 24.0 months (range, 0 to 58.0 months).

## Discussion

The goal of this retrospective study was to determine if increased serum SDMA concentrations are detected in cats with kidney stones. We found that 92% of cats in the current study with kidney stones had increased serum SDMA concentrations at the time of diagnosis, whereas 17% were azotemic at the time of diagnosis (42% were azotemic at some time prior to death). Therefore, we conclude than using serum SDMA as a biomarker for reduced kidney function allows earlier detection of kidney stones compared with measurement of serum creatinine. Of clinical importance, if serum SDMA concentrations are increased in mid-to-older aged cats, especially with USG > 1.035, then further imaging studies are warranted to evaluate for the presence of kidney stones.

Antemortem, kidney stones are usually found on abdominal radiographs and confirmed by ultrasonography [9]. In cats, kidney stones could obstruct the renal pelvis or ureter, predispose to pyelonephritis, or result in compressive injury of the renal parenchyma leading to progressive CKD [9]. It was formerly thought that nonobstructive kidney stones had no effect on mortality rate or rate of CKD progression in cats [10]. In fact, nonobstructive kidney stones are usually not treated unless they migrate into the ureter and cause ureteral obstruction [11]. However, because serum SDMA is elevated in cats with kidney stones, we conclude that nonobstructive kidney stones are associated with CKD and a reduction in GFR. Recent evidence has also shown a consistent relationship between nephrolithiasis and loss of kidney function in human patients [12–14].

The upper reference limit for SDMA in cats (<14 μg/dL) represents approximately 24% decrease from median GFR in healthy cats [6]. Serum SDMA increased in cats with CKD before serum Cr by a mean of 17.0 months (range, 1.5–48 months) [6]. In this study, the mean time that SDMA concentrations were increased above the upper reference limit before serum Cr concentrations were increased was 26.9 months (range, 0 to 60 months). Approximately 70% of cats with kidney stones in this colony were not discovered until necropsy even though cats are routinely monitored by clinical pathology assessments. However, only recently have serum SDMA concentrations been routinely included on serum chemistry panels and in this study they were determined retrospectively from banked serum. In the future, an annual blood test that detects increased serum SDMA concentration would suggest a need to perform radiographic imaging to check for the presence of kidney stones, before kidney function declines further and serum Cr concentrations increase.

Symmetric dimethylarginine is produced when arginine residues in proteins are post-translationally modified by methylation. Subsequent degradation of proteins containing methylated arginines yields individual methylated arginine amino acids. Free methylarginines are released into the cytosol following proteolysis, and then enter the blood circulation. Symmetric dimethylarginine has been shown to be a filtration marker that is excreted primarily (≥ 90%) by renal clearance without any metabolic degradation pathway, unlike ADMA being degraded primarily to dimethylamine and citrulline (≥80%) after metabolism by dimethylarginine dimethylaminohydrolase and only a small amount of intact ADMA (≤20%) being eliminated by the kidneys [15, 16]. Because serum SDMA is eliminated by the kidneys, plasma concentrations are affected by changes in GFR. Measurement of GFR is the gold standard method for estimating renal function. A meta-analysis of 18 studies in humans showed that serum SDMA concentration correlates highly with the GFR [17]. We have shown that serum SDMA correlates with GFR in cats [6, 7] as well as in dogs [18]. Furthermore, serum SDMA concentrations are not affected by lean body mass in cats [7] or in dogs [19]. Thus, cats with kidney stones may have early compromised renal function with increased SDMA and nonazotemia, or more advanced renal dysfunction with azotemia.

In our study, approximately 90% of kidney stones were calcium oxalate monohydrate, which is consistent with the findings of others [2]. Medical (dietary) dissolution is not possible for the majority of kidney stones [9]. However, knowing they were present would allow for periodic monitoring by urinalysis, urine culture, and imaging studies, as well as treatment according to guidelines for IRIS stage 1 CKD with increased SDMA and normal serum Cr. In addition, clinical trials could be undertaken for dissolution studies.

In humans, symptomatic urolithiasis has a life-time prevalence ranging from 5.9 to 12.5% in men and 3.7 to 5% in women [20–22] with the principal crystal component in the majority (70–80%) of these stones consisting of calcium oxalate monohydrate or dihydrate [23]. Familial aggregation patterns and more recently, studies in twins, have suggested a high degree of heritability [24–26]. Of the multiple metabolic abnormalities identified in these patients, even mild increases in the urinary excretion of oxalate, detected in about 20%, [27] have been shown to significantly impact calcium oxalate supersaturation and consequently, calcium oxalate crystal mass and the risk of calcium oxalate stone formation [28, 29].

The lifespan of cats with kidney stones (mean, 12.5 years) was shortened compared with that of healthy geriatric control cats (mean, 15.2 years). Furthermore, the lifespan of control cats was consistent with the lifespan of cats in the colony in general (age of death of the last 425 cats that died was mean, 14.5 years (range 1.6 to 20.0 years; unpublished results). The slightly reduced age at death of the colony population compared with control cats is because control cats were chosen as healthy geriatric adults, whereas some cats in the colony population died before reaching the age control cats were when selected. Our findings disagree with the findings of Ross et al. [10] who found in 14 cats with stage 2 or stage 3 CKD (7 with nephroliths and 7 without) that the presence of kidney stones was not associated with increased mortality rate or disease progression. The difference in control groups most likely explains the difference between studies, as control cats in our study did not have CKD. Also, not all our cats with nephrolithiasis were stage 2 or stage 3 CKD (only 42% had increased serum Cr at some point prior to death).

As a group, the 12 cats with kidney stones diagnosed antemortem differed from healthy geriatric control cats in that serum SDMA, Cr, and urea nitrogen concentrations were higher in cats with kidney stones, and USG was lower. These differences were more difficult to discern for individual cats with nephroliths because of overlapping concentration ranges.

Serum SDMA can now be measured routinely on clinical chemistry panels submitted to IDEXX Laboratories, Inc. at no additional cost. Serum SDMA is a more reliable and sensitive blood test for detecting underlying kidney dysfunction than serum creatinine. Finding increased serum SDMA concentrations suggests a need to perform radiographic imaging to check for the presence of kidney stones, before kidney function declines further and serum Cr concentrations increase. Measuring serum Cr is of little diagnostic help for detecting kidney stones. If kidney stones can be diagnosed more frequently antemortem, it will be easier to design controlled clinical studies to evaluate medical dissolution therapies in cats with kidney stones.

## Abbreviations

CKD: chronic kidney disease
Cr: creatinine
GFR: glomerular filtration rate
LC-MS: liquid chromatography-mass spectroscopy
SDMA: symmetric dimethylarginine
UPC: urine protein: creatinine
USG: urine specific gravity
